# Prenatal Exposure to Residential Air Pollution and Infant Mental Development: Modulation by Antioxidants and Detoxification Factors

**DOI:** 10.1289/ehp.1103469

**Published:** 2011-08-25

**Authors:** Mònica Guxens, Inmaculada Aguilera, Ferran Ballester, Marisa Estarlich, Ana Fernández-Somoano, Aitana Lertxundi, Nerea Lertxundi, Michelle A. Mendez, Adonina Tardón, Martine Vrijheid, Jordi Sunyer, on behalf of the INMA (INfancia y Medio Ambiente) Project

**Affiliations:** 1 Center for Research in Environmental Epidemiology (CREAL), Barcelona, Catalonia, Spain; 2 Hospital del Mar Research Institute (IMIM), Barcelona, Catalonia, Spain; 3 CIBER Epidemiologia y Salud Pública (CIBERESP), Spain; 4 Division of Environment and Health, Center for Public Health Research-CSISP, Valencia, Spain; 5 School of Nursing, University of Valencia, Valencia, Spain; 6 University of Oviedo, Oviedo, Spain; 7 Campus de Bizkaia, Universidad del País Vasco Leioa, Basque Country, Spain; 8 Instituto de Investigación Sanitaria de Euskadi (BIODONOSTIA), Donostia, Basque Country, Spain; 9 Campus de Gipuzkoa, Universidad del País Vasco, Donostia, Basque Country, Spain; 10 Pompeu Fabra University, Barcelona, Catalonia, Spain

**Keywords:** aromatic hydrocarbons, breast-feeding, child development, cognition, environmental pollution, fruit, intelligence, nitrogen dioxide, vegetables, vitamin D

## Abstract

Background: Air pollution effects on children’s neurodevelopment have recently been suggested to occur most likely through the oxidative stress pathway.

Objective: We aimed to assess whether prenatal exposure to residential air pollution is associated with impaired infant mental development, and whether antioxidant/detoxification factors modulate this association.

Methods: In the Spanish INfancia y Medio Ambiente (INMA; Environment and Childhood) Project, 2,644 pregnant women were recruited during their first trimester. Nitrogen dioxide (NO_2_) and benzene were measured with passive samplers covering the study areas. Land use regression models were developed for each pollutant to predict average outdoor air pollution levels for the entire pregnancy at each residential address. Maternal diet was obtained at first trimester through a validated food frequency questionnaire. Around 14 months, infant mental development was assessed using Bayley Scales of Infant Development.

Results: Among the 1,889 children included in the analysis, mean exposure during pregnancy was 29.0 μg/m^3^ for NO_2_ and 1.5 μg/m^3^ for benzene. Exposure to NO_2_ and benzene showed an inverse association with mental development, although not statistically significant, after adjusting for potential confounders [β (95% confidence interval) = –0.95 (–3.90, 1.89) and –1.57 (–3.69, 0.56), respectively, for a doubling of each compound]. Stronger inverse associations were estimated for both pollutants among infants whose mothers reported low intakes of fruits/vegetables during pregnancy [–4.13 (–7.06, –1.21) and –4.37 (–6.89, –1.86) for NO_2_ and benzene, respectively], with little evidence of associations in the high-intake group (interaction *p*-values of 0.073 and 0.047). Inverse associations were also stronger in non-breast-fed infants and infants with low maternal vitamin D, but effect estimates and interactions were not significant.

Conclusions: Our findings suggest that prenatal exposure to residential air pollutants may adversely affect infant mental development, but potential effects may be limited to infants whose mothers report low antioxidant intakes.

Ambient air pollution is a global public health threat. Cardiorespiratory effects and related mechanisms have been extensively investigated (Götschi et al. 2008; Sun et al. 2010). However, little is known about possible neurological effects, because preliminary evidence is limited (Sunyer 2008). Animal studies have shown that air pollutants might reach the brain directly via the olfactory bulb and may themselves be proinflammatory (Block and Calderon-Garciduenas 2009). Moreover, air pollutants seem to induce oxidative stress in the lung and cause chronic respiratory tract and systemic inflammation that may lead to brain inflammation by increasing levels of circulating cytokines (Block and Calderon-Garciduenas 2009).

Maturation of the cortex during the first years of life is intensive (Grandjean and Landrigan 2006). This period of life is considered an important window for brain development, because the brain’s plasticity decreases with age, and susceptibility to environmental insults is elevated (Grandjean and Landrigan 2006). Nine studies concerning neuropsychological effects of air pollution on children have been published to date (Calderon-Garciduenas et al. 2008; Edwards et al. 2010; Freire et al. 2010; Perera et al. 2006, 2008, 2009; Siddique et al. 2011; Suglia et al. 2008; Tang et al. 2008; Volk et al. 2011; Wang et al. 2009). However, larger and more detailed research is needed given the potential worldwide impact of these findings. Furthermore, some researchers have pointed out that, at least in some countries, disadvantaged groups are more likely to live in areas of greater air pollution (Brulle and Pellow 2006). Consequently, health effects of air pollution exposure should be carefully separated from the potential confounding role of socioeconomic inequalities. Among the factors related to health disadvantage and vulnerability, high intakes of antioxidant nutrients has been proposed as an important potential modifier of air pollution impairment (Romieu et al. 2008; Villarreal-Calderon et al. 2010). Thus, we hypothesized that levels of different antioxidants and detoxification factors during pregnancy and the first year of life may modulate the potential negative effect of air pollutants on mental development. Fruits and vegetables are a rich source of antioxidant nutrients (Balsano and Alisi 2009), and breast milk has a high content of long-chain polyunsaturated fatty acids and other micronutrients that may reduce inflammation and oxidative stress (Rodriguez-Palmero et al. 1999). Moreover, a beneficial role of vitamin D in the developing brain through antioxidant and detoxification mechanisms has recently been postulated (Buell and Dawson-Hughes 2008).

Therefore, the purpose of the present study was to assess whether residential air pollution exposure during pregnancy adversely affects mental development during the second year of life, and whether antioxidant and detoxification factors modulate this association, in a population-based birth cohort in which air pollution exposures were not associated with social class determinants (Vrijheid et al. 2010).

## Materials and Methods

*Study design and participants.* Population-based birth cohorts were established as part of the INMA (INfancia y Medio Ambiente; Environment and Childhood) Project in several regions of Spain following a common protocol (Guxens et al. 2011). This analysis uses the INMA cohorts of Valencia, Sabadell (Catalonia), Asturias, and Gipuzkoa (Basque Country) established between 2003 and 2008. Pregnant women were enrolled during the first trimester of pregnancy at public primary health care centers or public hospitals, depending on the region, providing they fulfilled the inclusion criteria (≥ 16 years of age, intention to deliver at the reference hospital, no problems of communication, singleton pregnancy, no assisted conception); 99.5% of Spaniards have public health insurance, and 70–90% of women use public health services during pregnancy (Regidor et al. 2008). Of all women invited, 56% agreed to participate. Women were then followed throughout pregnancy. Their children were followed from birth through 2 years of age. Informed consent was obtained from all participants, and the study was approved by the hospital ethics committees in each participating region.

*Child mental development test.* Children’s mental development was assessed at around 14 months of age (range, 11–23 months) using the Bayley Scales of Infant Development (Bayley 1993). The Bayley mental development scale consists of 163 items that assess age-appropriate mental development, including performance abilities, memory, and early language skills. All testing was done in the health care center in the presence of the mother, by 12 specially trained psychologists. Psychologists were not aware of any exposure information. To limit interobserver variability, we applied a strict protocol, including training sessions where interobserver differences were quantified and three sets of quality controls (interobserver reliability tests) undertaken during the fieldwork. The interrater reliability, estimated by intraclass correlation, was 0.90, and Cronbach’s alpha coefficient, a measure of internal consistency, was 0.70. Tests were performed on 2,213 children who attended the visit. Eighteen children were excluded because of specific pathologies, and 144 were excluded because their test results were of uncertain quality due to less than optimal cooperation. Raw scores were standardized for child’s age in days at test administration using a parametric method for the estimation of age-specific reference intervals. The parameters of the distribution were modeled as a fractional polynomial function of age and estimated by maximum likelihood. Residuals were then standardized to a mean of 100 points with a standard deviation of 15 points to homogenize the scales.

*Assessment of air pollution exposure.* A complete description of the methodology on exposure modeling has been reported previously (Aguilera et al. 2008; Estarlich et al. 2011; Fernández-Somoano et al. 2011; Iñiguez et al. 2009). Briefly, ambient concentrations of nitrogen dioxide (NO_2_) and benzene were repeatedly measured with passive samplers distributed over the study areas according to geographic criteria, taking into account the expected pollution gradients and the expected number of births. The samplers measured pollutants levels using radial symmetry (Radiello®; Fundazione Salvatore Maugeri, Padua, Italy) and remained exposed during various sampling periods of 7 days each. Land use regression models were used to predict NO_2_ and benzene levels at women’s residential addresses, taking into account any residential changes during pregnancy. Geographic information system data derived in ArcGIS version 9.1 (ESRI, Redlands, CA, USA) (traffic as measured by vehicle density, distance of the home from a main road, land use, and altitude) were used to obtain predictor variables. Spatial estimates were temporally adjusted using serial records from the network of monitoring stations covering the study areas, to obtain estimates for each woman’s specific pregnancy period. Finally, an average exposure level over the whole pregnancy period was calculated.

*Antioxidant/detoxification variables.* Information on maternal diet was obtained in the first trimester of pregnancy using a 101-item semiquantitative validated food frequency questionnaire (Vioque et al. 2007). Women were asked to report usual intakes since the start of pregnancy using reference portion sizes and nine frequency categories ranging from “never/less than once per month” to “6 or more times per day.” The food frequency questionnaire included 10 fruit items (oranges; fresh-squeezed orange juice; bananas; apples or pears; peaches, nectarines, or apricots; watermelon or melon; grapes; plums; kiwis; and olives) and 12 vegetable items (spinach; cabbage, cauliflower, or broccoli; lettuce or endive; tomatoes; onions; carrots or squash; green beans; eggplant or zucchini; bell peppers; artichokes; asparagus; and garlic). Reported intakes of each item were converted to estimated daily frequencies using the midpoint of each category (e.g., a frequency of 1–3 times/month was converted to 2 times/30 days or 0.067 times/day). All items were summed to estimate overall fruit and vegetables intakes. Fruit and vegetable intakes were categorized in two groups: low tertile (≤ 405 g/day) versus medium/high tertile (> 405 g/day).

Detailed information about child feeding through the second year of life was collected from mothers by interviewer-administered questionnaires. Breast-feeding was defined as receiving any breast milk, regardless of supplementation with food or other liquids, including nonhuman milk. Duration of breast-feeding was categorized into three groups: children who were never breast-fed, children who were breast-fed for a short time period (< 6 months), and long-term breast-fed children (≥ 6 months).

Plasma 25-hydroxyvitamin D_3_ [25(OH)D] reflects contributions from all sources of vitamin D (i.e., diet and sun exposure) and is considered the best circulating biomarker of vitamin D metabolic status. A single maternal fasting blood specimen was drawn during pregnancy (mean ± SD, 13.4 ± 1.7 weeks of gestation). Samples were processed immediately and stored at –70°C to –80°C until analysis. Levels of maternal plasma vitamin D were determined by high-performance liquid chromatography using a BioRAD (Madrid, Spain) kit according to Clinical and Laboratory Standard Institute (NCCLS) protocols. Because 25(OH)D levels are known to vary over the course of the year, we used season-specific cut points in the analyses to deal with season of blood drawn (spring, summer, fall, and winter). Season-specific tertiles were constructed: low (< 22.1, < 30.7, < 25.2, and < 21.0 ng/mL for spring, summer, fall, and winter, respectively), medium (22.1–32.0, 30.7–39.3, 25.2–33.7, and 21.0–30.7 ng/mL, respectively), and high (> 32.0, > 39.3, > 33.7, and > 30.7 ng/mL, respectively).

*Other parental and child variables.* Questionnaires during the first trimester of pregnancy obtained information about the highest achieved level of parental education, parental occupation, parental country of origin, parental age, maternal height and prepregnancy weight, parity, and marital status. We defined parental social class from the maternal or paternal occupation during pregnancy based on the highest social class, using a widely used Spanish adaptation of the international ISCO88 (International Standard Classification of Occupations) coding system (Instituto Nacional de Estadística 1994). Maternal tobacco and alcohol use, second-hand smoke at home or in the workplace, use of a gas stove at home during pregnancy, and annoyance due to noise at home were collected through questionnaires during the third trimester. Maternal levels of hemoglobin and thyroid-stimulating hormone were analyzed in maternal serum extracted in the first trimester. Maternal cotinine levels were measured in maternal urine collected in the third trimester of pregnancy, using the Cotinine Micro-Plate EIA Kit (Ora Sure Technologies, Inc., Bethlehem, PA, USA). Total lead levels were analyzed in cord blood, using thermal decomposition, amalgamation, and atomic absorption spectrometry with a detection limit of 2 μg/L. Almost all cohort members had lead levels below the limit of detection (95.4%). Information related to the child’s gestational age, sex, anthropometric measures, type of delivery, and Apgar score at birth was obtained from clinical records. In a subsequent interview when the child was 14 months of age (range, 11–23 months), information on changes in residence since the third trimester of pregnancy, main caregiver, nursery attendance, maternal working status, use of gas stove at home, and child second-hand smoke exposure was collected. All questionnaires were administered face to face by trained interviewers.

*Statistical analysis.* Statistical analyses included all subjects with complete data on mental development and air pollution (*n* = 2,138). Preterm births (< 37 weeks of gestation; *n* = 81), children with unknown gestational age (*n* = 12), children with specific pathologies (*n* = 18), and children with low-quality neuropsychological tests (*n* = 138) were excluded. Among the included subjects (*n* = 1,889), multiple imputation of missing values for the covariables was performed using chained equations where 20 completed data sets were generated and analyzed using the standard combination rules for multiple imputation [see Supplemental Material, Tables 1, 2 (http://dx.doi.org/10.1289/ehp.1103469)] (Spratt et al. 2010; Sterne et al. 2009).

**Table 1:** Distribution of characteristics of interest by region.

Characteristic	Overall (*n* = 1,889)	Valencia (*n* = 608)	Sabadell (*n* = 471)	Asturias (*n* = 345)	Gipuzkoa (*n* = 465)
Mental development score	99.9 ± 15.2		100.7 ± 15.5		99.1 ± 14.6		101.6 ± 15.6		98.5 ± 15.2
Age at mental development assessment (months)	14.8 ± 2.6		12.4 ± 0.8		14.6 ± 0.7		19.4 ± 1.3		14.5 ± 0.8
NO_2_ exposure during pregnancy (μg/m^3^)	29.0 ± 11.2		36.8 ± 11.0		32.1 ± 8.8		23.2 ± 7.1		20.1 ± 6.5
Benzene exposure during pregnancy (μg/m^3^)	1.5 ± 0.9		2.2 ± 0.6		0.8 ± 0.3		2.3 ± 1.3		1.0 ± 0.3
Maternal fruit and vegetable consumption, first trimester*a*									
Low tertile (≤ 405 g/day)	33.5		39.8		33.0		30.5		27.6
Medium/high tertile (> 405 g/day)	66.5		60.2		67.0		69.5		72.4
Breast-feeding duration									
None	14.7		16.0		6.7		29.0		10.5
< 6 months	40.5		39.9		42.6		43.2		36.9
≥ 6 months	44.8		44.1		50.7		27.8		52.6
Maternal circulating vitamin D levels*b*									
Low	33.6		22.7		40.4		40.0		35.7
Medium	33.0		34.0		27.1		35.5		35.9
High	33.4		43.3		32.5		24.5		28.4
Parental social class*c*									
I/II managers/technicians	32.8		23.5		32.0		32.6		45.8
III skilled manual/nonmanual	25.8		28.1		28.7		24.1		20.9
IV/V semiskilled/unskilled	41.4		48.4		29.3		43.3		33.3
Maternal education level									
Primary or less	22.8		31.7		26.3		15.6		12.7
Secondary	41.2		43.3		42.5		43.2		35.7
University degree	36.0		25.0		31.2		41.2		51.6
Values are percentages for categorical variables and mean ± SD for continuous variables. **a**Low versus medium/high tertile of maternal fruit and vegetable consumption. **b**Season-specific tertiles of maternal circulating vitamin D levels (see “Materials and Methods”). **c**Social class data from Instituto Nacional de Estadística (1994).									

**Table 2:** Adjusted association for a doubling in NO_2_ and benzene levels during pregnancy and infant mental development.*a*

Effect per doubling in NO_2_ levels during pregnancy					Effect per doubling in benzene levels during pregnancy				
Characteristics	*n*	β-Coefficient (95% CI)	*p*-Value*b* interaction	*n*	β-Coefficient (95% CI)	*p*-Value*b* interaction
Maternal fruit and vegetable consumption, first trimester*c*												
Low tertile (≤ 405 g/day)		627		–4.13 (–7.06, –1.21)				613		–4.37 (–6.89, –1.86)		
Medium/high tertile (> 405 g/day)		1,249		0.25 (–3.63, 4.12)		0.073		1,228		–0.60 (–3.66, 2.46)		0.047
Breast-feeding duration												
None		270		–3.47 (–7.82, 0.98)				262		–3.53 (–7.15, 0.08)		
< 6 months		745		–0.71 (–4.06, 2.65)		0.274		729		–2.42 (–6.08, 1.25)		0.133
≥ 6 months		826		–0.61 (–2.97, 1.75)		0.196		816		0.05 (–2.22, 2.31)		0.070
Maternal circulating vitamin D levels*d*												
Low		607		–2.49 (–6.87, 1.89)				594		–2.34 (–5.34, 0.04)		
Medium		597		–0.55 (–3.48, 2.39)		0.571		586		–0.26 (–2.87, 2.38)		0.292
High		605		–0.11 (–2.72, 2.49)		0.268		597		–0.34 (–3.17, 1.75)		0.241
Parental social class*e*												
I/II managers/technicians		619		–1.02 (–5.23, 3.19)				605		–1.00 (–3.32, 1.32)		
III skilled manual/nonmanual		486		0.16 (–3.79, 4.11)		0.565		476		–0.62 (–3.55, 2.32)		0.727
IV/V semiskilled/unskilled		783		–1.57 (–3.76, 0.62)		0.725		772		–2.25 (–5.19, 0.68)		0.614
Maternal education level												
Primary or less		429		–0.90 (–6.54, 4.73)				425		–2.58 (–7.56, 2.41)		
Secondary		776		–2.22 (–5.13, 0.70)		0.795		765		–1.46 (–4.95, 2.03)		0.665
University degree		679		–0.72 (–3.93, 2.49)		0.630		659		–1.22 (–3.45, 1.01)		0.797
Each cell represents a random-effect model by region from a meta-analysis. **a**Adjusted for psychologist, child’s sex, child’s age at mental development assessment, maternal education, maternal age, maternal height and prepregnancy body mass index, maternal alcohol use during pregnancy, maternal large fatty and lean fish consumption at first trimester, season-specific maternal circulating vitamin D level at pregnancy, use of gas stove at home during pregnancy, and number of siblings at birth. **b***p*-Values for interaction terms based on region-specific interactions that were combined using meta-analysis. **c**Low versus medium/high tertile of maternal fruit and vegetable consumption. **d**Season-specific tertiles of maternal circulating vitamin D levels (see “Materials and Methods”). These models were not adjusted for maternal vitamin D levels at pregnancy. **e**Social class data from Instituto Nacional de Estadística (1994).												

We used generalized additive models to assess the linearity of the relationship between NO_2_ and benzene and infant mental development by graphical examination and likelihood ratio tests. Benzene was not linearly related with infant mental development (*p*-value for gain in linearity = 0.059), and benzene and NO_2_ were therefore log_2_ transformed. Multivariable linear regression models were performed to examine the relationship between log-transformed NO_2_ and benzene and infant mental development as continuous normal variables.

First, to assess the effect of potential mediators and confounders on coefficients for associations with each air pollution variable, a series of models were run to assess the effect of additionally adjusting (forward selection) and eliminating (backward elimination) one by one each of the individual parental and child characteristics of interest. Separate models were run for each region. Final multivariable-adjusted models simultaneously incorporated all covariates either associated with mental scores (*p* < 0.20) [see Supplemental Material, Figure 1 (http://dx.doi.org/10.1289/ehp.1103469)] or that modified the coefficient for the air pollutant variables by > 15% in any region.

**Figure 1:**
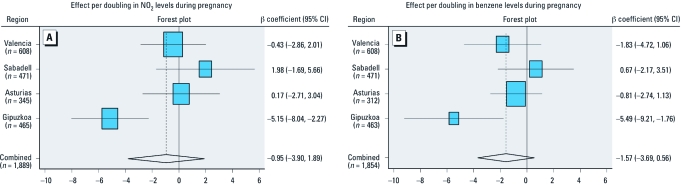
Region and summary risk estimates (β-coefficient and 95% CI) for a doubling in NO_2_ levels (*A*) and benzene levels (*B*) during pregnancy and infant mental development, adjusted for psychologist, child’s sex, child’s age at mental development assessment, maternal education, maternal age, maternal height and prepregnancy body mass index, maternal alcohol use during pregnancy, maternal large fatty and lean fish consumption at first trimester, season-specific maternal circulating vitamin D level at pregnancy, use of gas stove at home during pregnancy, and number of siblings at birth. (*A*) Test for heterogeneity: *Q* = 11.314 on three degrees of freedom (*p* = 0.010). (*B*) Test for heterogeneity: *Q* = 7.084 on three degrees of freedom (*p* = 0.068). The size of the markers for each β-coefficient represents the relative weight that each region contributed to the summary regression slope.

In a second step, we performed a meta-analysis using random-effects models combining the estimates in each region of the association between each air pollutant and infant mental development. Exposure–response slopes derived for each region were plotted together with the summary slope from the meta-analysis using forest plots of β-coefficients with 95% confidence intervals (CIs). We assessed heterogeneity in risk estimates using the *Q*-test.

In a third step, effect modification of air pollution associations by levels of antioxidants and detoxification factors (fruits and vegetables, breast-feeding, circulating vitamin D), as well as by socioeconomic variables (parental social class and maternal education), was assessed using stratified analysis and interaction terms. Models for each region were fitted stratifying by or including an interaction term for each variable of interest. Results for each stratum were then combined using meta-analysis.

We performed various sensitivity analyses to assess the robustness of our results. First, multivariable-adjusted models were further adjusted for other environmental pollutants and socioeconomic variables to minimize the likelihood of residual confounding. Moreover, because fetal growth restriction might be an intermediate factor of the assessed relation (Slama et al. 2008), the possible mediation of birth outcomes was tested by including them in the multivariable-adjusted models. We also repeated the multivariable-adjusted models in specific population subgroups (including country of origin, social class, parental education, and subjects with stable residences) to reduce residual confounding. Finally, to assess the consistency of evidence for effect modification, we examined the stratified analyses and the interaction terms in models run separately for each region and in pooled analyses, excluding the Gipuzkoa region because heterogeneity across regions was mainly attributable to this region. Statistical analyses were conducted using STATA (version 10.1; StataCorp, College Station, TX, USA). Statistical tests of hypotheses were two tailed, with significance set at *p* < 0.05.

## Results

A total of 2,644 pregnant women were recruited during the first trimester of pregnancy [see Supplemental Material, Figure 2 (http://dx.doi.org/10.1289/ehp.1103469)]. A total of 2,505 (94.7%) children were enrolled at birth, 2,348 (88.8%) were assessed in the second year of life, and 2,138 (80.9%) had complete mental development and air pollution data. Our analysis is based on 1,889 children after some exclusions (preterm births, children with unknown gestational age, children with specific pathologies, and children with low-quality neuropsychological tests). No statistically significant differences between the 1,889 children in the analysis sample and the original 2,505 were observed in terms of maternal alcohol consumption during pregnancy, maternal prepregnancy body mass index, paternal education and age, parental country of origin, or number of siblings at birth [see Supplemental Material, Table 3 (http://dx.doi.org/10.1289/ehp.1103469)]. However, children not included had lower parental social class, lower maternal education, higher maternal smoking use, higher proportion of one-parent families, younger mothers, lower gestational age, lower birth weight, higher proportion of boys, and shorter breast-feeding duration.

Among the 1,889 children included in the analysis, mean air pollution exposure during pregnancy was 29.0 μg/m^3^ (range, 20.1–36.8 across regions) for NO_2_ and 1.5 μg/m^3^ (range, 0.8–2.3) for benzene (Table 1). Exposure to NO_2_ and benzene was inversely associated with mental development, although relationships were not statistically significant, in unadjusted models and after adjusting for a large array of potential confounders (Figure 1). Among regions, main effect estimates were significant for Gipuzkoa only [β (95% CI) = –5.15 (–8.04, –2.27) and –5.49 (–9.21, –1.76), respectively, for a doubling of each compound].

When we stratified by antioxidant and detoxification variables, we found a strong and significant inverse relationship between NO_2_ and benzene and mental development score among infants with low maternal intakes of fruits and vegetables during the first trimester of pregnancy, but little evidence of an association among infants whose mothers had higher intakes (interaction *p*-values = 0.073 and 0.047, respectively) (Table 2). Inverse associations with NO_2_ and benzene were also stronger in infants that were not breast-fed and for infants whose mothers had low circulating vitamin D during pregnancy than in other infants, although stratum-specific estimates and interactions were not significant (Table 2). Associations between air pollution and infant mental development were not significantly different according to parental social class or education level (*p* > 0.20) (Table 2).

Region-specific effect estimates were relatively homogeneous for infants whose mothers had low fruit and vegetable intakes (heterogeneity *p*-values of 0.3 and 0.7 for associations with NO_2_ and benzene, respectively) compared with the high-intake group (heterogeneity *p*-values of 0.002 and 0.02) [Supplemental Material, Figure 3 (http://dx.doi.org/10.1289/ehp.1103469)]. However, excluding Gipuzkoa, which was the only region where NO_2_ and benzene were significantly associated with lower mental development scores in the group with high fruit and vegetable intake, did not have a meaningful effect on interactions between air pollutants and antioxidants, detoxification factors, or socioeconomic variables (Supplemental Material, Table 4).

In the group with low maternal fruits and vegetable consumption, results did not change meaningfully after additionally adjusting for noise annoyance, maternal smoking during pregnancy, maternal cotinine levels during pregnancy, parental social class, and parental country of origin, considered to be potential mediating factors (data not shown). Additionally adjusting for birth weight, birth height, birth head circumference, and gestational age also did not meaningfully change the estimated coefficients (data not shown). Similarly, when we restricted the analysis to families without residential changes between the third trimester of pregnancy and the age at developmental testing [β (95% CI) = –2.89 (–5.91, 0.13) and –3.49 (–6.34, –0.63), respectively, for a doubling of each compound] or to women who spent > 15 hr/day at home during pregnancy [β (95% CI) = –4.38 (–7.72, –1.04) and –4.01 (–6.96, –1.07), respectively, for a doubling of each compound], relations remained consistent. Results were also comparable to those shown for the main models when we repeated these sensitivity analyses among infants who were not breast-fed, in the low maternal vitamin D group, or in the high fruit-and-vegetable intake group (data not shown).

## Discussion

In this study conducted in several regions of Spain, adverse effects of air pollutants on infant mental development in the second year of life were observed among subjects with low exposure to maternal consumption of fruits and vegetables. The protective effect of other factors was also tested showing a nonsignificant inverse association in non-breast-fed infants and infants with low maternal vitamin D. These findings were very stable in several sensitivity analyses. In Gipuzkoa, however, there was an overall adverse effect of prenatal exposure to residential air pollution on mental development scores, regardless of exposure to these protective factors. An important advantage of this study was that air pollution was not clearly related with social class indicators (Vrijheid et al. 2010), and there was little or no evidence of confounding or modification by social class.

NO_2_ and benzene are considered markers of toxic air pollutants rather than potential causative agents themselves. A limitation of our study was that only NO_2_ and benzene were measured, instead of ultrafine particulate matter (PM)—particularly the trace metal content—that seems to be the most neurotoxic component of residential air pollution (Block and Calderon-Garciduenas 2009). A second limitation was that noise annoyance during pregnancy was collected by a self-reported scale rather than by a direct measure of noise levels. Both air and noise pollution are associated with motor vehicle traffic, and recent studies have reported possible associations between children’s cognition and road traffic noise (Clark and Stansfeld 2007). Although our analyses were adjusted for noise annoyance, some residual confounding may remain. Another limitation was that parental intelligence, an important determinant of infant mental development, was not evaluated. Parental education level and social class did not confound or modify the associations, but their inclusion in the model cannot completely eliminate possible residual confounding by parental intelligence. Finally, not all children initially recruited at birth (*n* = 2,505) were included in the analysis sample (25% loss to follow-up; *n* = 616), and loss to follow-up was related to lower social status. However, the inclusion in the analysis of a large set of variables related to participation, and the consistent results obtained across strata of various socioeconomic factors, suggests that nonresponse is unlikely to have biased the results.

A major strength of this study is the assessment of air pollution at the individual level using the most robust exposure assessment methods currently available for use in large observational studies (Aguilera et al. 2008). Assessments based on residential location should very precisely reflect exposure in early life. Moreover, associations persisted after taking into account time–activity patterns during pregnancy, such as hours spent at home. Additional strengths were the time window when exposure was assessed—the prenatal period—as well as the prospective design. Although pre- and postnatal exposures cannot be disentangled given that most children remained in the same residence in both periods, exposures during the postnatal period could also play a role in the mental developmental effects observed. Finally, the availability of extensive data on nutrition permitted us to explore potential protective mechanisms suggested in laboratory experiments exploring the role of air pollutants in the brain (Block and Calderon-Garciduenas 2009).

Our results suggest that higher consumption of fruits and vegetables during pregnancy may have mitigated an adverse effect of ambient air pollutants on infant mental development in our study population, whereas longer breast-feeding duration and higher maternal circulating vitamin D levels at pregnancy appeared to have less of an effect on associations. Although maternal fruit and vegetable consumption was not related to maternal socioeconomic status, this was not the case for breast-feeding duration and maternal circulating vitamin D levels (data not shown). Although some residual confounding may remain, our results support the hypothesized biological mechanisms through which air pollutants may affect mental development, mainly related to neuroinflammation and lipid peroxidation through oxidative stress in the brain (Block and Calderon-Garciduenas 2009). Consistent with our results, Villarreal-Calderon et al. (2010) also found a sustained dorsovagal complex inflammation in mice exposed to Mexico city air, which were mitigated by administration of dark chocolate, which is rich in polyphenols, potent antioxidants. Evidence of a protective effect of antioxidant intake on associations between air pollution and respiratory outcomes has also been found in several other studies, as reviewed by Romieu et al. (2008).

The fact that negative associations between air pollution and mental development were observed among all infants from Gipuzkoa—rather than only those with potentially increased susceptibility—may be partly attributable to a more toxic mixture of air pollutants in that region. In Valencia and Sabadell, trace metals contribute < 0.5% to fine particulate matter mass (lead, 5.9–22.4 μg/m^3^; manganese, 2.4–13.1 μg/m^3^), similar to other European cities (Rivas-Lara 2008; Viana et al. 2008) [see Supplemental Material, Table 5 (http://dx.doi.org/10.1289/ehp.1103469)]. In contrast, Gipuzkoa, a region with high levels of industrial activity—mainly iron- and steelworks—has mean concentrations of trace metals in the fine PM fraction that greatly exceed those of other European cities [lead, 20.3–224.0 μg/m^3^; manganese, 10.2–124.0 μg/m^3^) (see Supplemental Material, Table 5) (Lertxundi et al. 2010). Fine PM and its components have not yet been measured in Asturias, which is also an industrial area with steelworks, glassworks, and chemical activity that could also have high levels of trace metals. However, because of geographic and meteorologic differences, there may be substantial disparities in dispersion patterns of various PM fractions in Gipuzkoa, where residents live primarily in narrow valleys where these particles may be trapped, as opposed to Asturias, which is a coastal and less mountainous region. Among trace metals, lead levels have previously been related with impaired infant neuropsychological development (Grandjean and Landrigan 2006). Although the Gipuzkoa region had high levels of lead in ambient air, lead was detected in a very low percentage of cord blood samples, and levels were similar across all four regions (see Supplemental Material, Table 5) (Llop et al. 2011). These results, along with the fact that adjustment for lead levels did not confound the results in our study, suggest that either other metals such as manganese, or the combined effect of different metals, could be involved in the underlying neuroinflammation caused by air pollution. We speculate that atmospheres with a high content of trace metals could adversely affect mental development even among those protected by a high antioxidant intake and/or detoxification factors.

## Conclusions

This study suggests that some antioxidant factors such as maternal consumption of fruits and vegetables during pregnancy may modulate adverse effects of air pollutants on infant mental development. The magnitude of the association observed in subjects with low levels of antioxidant nutrient intakes is not sufficient to have important clinical implications at the individual level but, given the ubiquity of residential air pollution exposure, does have implications for a strong population-level impact. Continued follow-up of our birth cohort will allow us to explore if effects of early air pollutant exposure on cognitive development appear to be stronger at older ages, as has been shown for other neurotoxicants, perhaps because of improved ability to assess cognitive function at older ages (Perera et al. 2006, 2009). Should the apparent protective role of high antioxidants intake be confirmed in other studies, effective prevention programs could be developed for pregnant women focused in part on the promotion of protective dietary practices, as well as on the reduction of air pollution exposure.

## Supplemental Material

(111 KB) PDF
