# Supplementary material for: Prenatal Exposure to Residential Air Pollution and Infant Mental Development: Modulation by Antioxidants and Detoxification Factors
Source: Environ Health Perspect. 2011 Aug 25;120(1):144–9. doi: 10.1289/ehp.1103469 (PMC3261939; doi:10.1289/ehp.1103469)
Supplement: (111 KB) PDF [file ehp.1103469.s001.pdf]

Supplementary material.

Prenatal Exposure to Residential Air Pollution and Infant Mental Development: Modulation by Antioxidants and Detoxification Factors

Mònica Guxens, Inmaculada Aguilera, Ferran Ballester, Marisa Estarlich, Ana Fernández-Somoano, Aitana Lertxundi, Nerea Lertxundi, Michelle A. Mendez, Adonina Tardón, Martine Vrijheid, and Jordi Sunyer on behalf of the INMA Project

Supplemental Material, Table 1. Child and parental characteristics of the study population by region………………………………………………………………………. p. 2

Supplemental Material, Table 2. Details of the imputation modelling……………….p. 5

Supplemental Material, Figure 1. Bivariate association (β coefficient and 95% of Confidence Interval) between study variables and infant mental development…......p. 6

Supplemental Material, Figure 2. Flowchart illustrating the main phases in the study………………………………………………………………………………………p. 7

Supplemental Material, Table 3. Differences on child and parental characteristics between participants included and those not included in the present analyse……… p. 8

Supplemental Material, Figure 3. Region and summary risk estimates (β coefficient and 95% of Confidence Interval) for a doubling in NO_2 and benzene levels during pregnancy and infant mental development by maternal fruits and vegetables consumption levels[a]…………………………………………………….....p. 9

Supplemental Material, Table 4. Adjusted association (β coefficient and 95% of Confidence Interval) for a doubling in NO_2 and benzene levels during pregnancy and infant mental development. Pooled analyses excluding Gipuzkoa cohort………p. 11

Supplemental Material, Table 5. Characteristics of each region……………………..p. 12

Supplemental Material, Table 1a. Child and parental characteristics of the study population by region

	Valencia (n=608)		Sabadell (n=471)		Asturias (n=345)		Gizpuzkoa (n=465)	
**Characteristics**	**n**	**Distribution**	**n**	**Distribution**	**n**	**Distribution**	**n**	**Distribution**
**Sex**								
Male	307	50.5	242	51.4	175	50.7	220	47.3
Female	301	49.5	229	48.6	170	49.3	245	52.7
Missings	0		0		0		0	
**Gestational age (weeks)**	608	39.8 (1.2)	471	39.9 (1.2)	345	39.7 (1.2)	465	39.9 (1.2)
Missings	0		0		0		0	
**Type of delivery**								
Vaginal	461	76.7	395	85.7	283	82.0	385	87.3
Cesarean	140	23.3	66	14.3	62	18.0	56	12.7
Missings	7		10		0		24	
**Birthweight (gr)**	608	3287 (448)	470	3269 (399)	340	3323 (421)	461	3324 (414)
Missings	0		1		5		4	
**Birthlength (cm)**	608	50.3 (2.0)	458	49.5 (1.8)	337	49.8 (1.9)	436	49.0 (1.8)
Missings	0		13		8		29	
**Birth cephalic perimeter (cm)**	607	34.2 (1.4)	459	34.3 (1.2)	337	34.3 (1.4)	436	24.8 (1.2)
Missings	1		12		8		29	
**Number of siblings at child's birth**								
0	341	46.1	274	58.4	216	62.6	265	57.0
1 or more	267	43.9	195	41.6	129	37.4	200	43.0
Missings	0		2		0		0	
**Breastfeeding duration**								
No	97	16.0	31	6.7	96	29.0	46	10.5
<6 months	242	39.9	198	42.6	143	43.2	162	36.9
≥6 months	267	44.1	236	50.8	92	27.8	231	52.6
Missings	2		6		14		26	
**Main caregiver through child 2nd year of life**								
Mother	474	78.5	212	47.2	171	57.6	191	43.7
Both parents with/without grandparents	61	10.1	119	26.4	80	26.9	168	38.4
Other combinations	69	11.4	119	26.4	46	15.5	78	17.9
Missings	4		21		48		28	
**Nursery attendance through child 2nd year of life**								
No	480	79.5	317	69.8	155	52.2	222	50.7
Yes	124	20.5	137	30.2	142	47.8	216	49.3
Missings	4		17		48		27	

Values are percentages for categorical variables and mean (SD) for continuous variables

Supplemental Material, Table 1b. Child and parental characteristics of the study population by region

	Valencia (n=608)		Sabadell (n=471)		Asturias (n=345)		Gizpuzkoa (n=465)	
	n	Distribution	n	Distribution	n	Distribution	n	Distribution
**Parental social class**								
I/II Managers/ Technicians	143	23.5	151	32.0	122	32.6	213	45.8
III/IV Skilled manual/ non-manual	171	28.1	135	28.7	83	24.1	97	20.9
V/VI Semi-skilled/ unskilled	294	48.4	185	39.3	149	43.3	155	33.3
Missings	0		0		1		0	
**Maternal education level**								
Primary or less	193	31.7	123	26.3	54	15.6	59	12.7
Secondary	263	43.3	199	42.5	149	43.2	165	35.7
University degree	152	25.0	146	31.2	142	41.2	239	51.6
Missings	0		3		0		2	
**Paternal education level**								
Primary or less	276	45.5	163	35.1	97	28.2	109	23.8
Secondary	230	37.9	201	43.2	149	46.2	232	50.5
University degree	101	16.6	101	21.7	88	25.6	118	25.7
Missings	1		6		1		6	
**Maternal age at child's birth (years)**	608	31.1 (4.4)	470	31.6 (4.2)	345	32.9 (4.2)	465	32.6 (3.5)
Missings	0		1		0		0	
**Paternal age at child’s birth (years)**	607	33.1 (4.9)	469	33.5 (4.8)	345	35.2 (5.1)	465	35.1 (4.5)
Missings	1		2		0		0	
**Parental country of birth**								
Spain	492	80.9	396	85.0	325	94.2	446	95.9
Foreign	116	19.1	70	15.0	20	5.8	19	4.1
Missings	0		5		0		0	
**Family status**								
Biparental	599	98.5	465	98.9	340	98.6	463	99.6
Monoparental	9	1.5	5	1.1	5	1.4	2	0.4
Missings	0		1		0		0	
**Smoking at 3rd trimestre**								
No	472	77.6	405	86.9	279	83.5	407	89.3
Yes	136	22.7	61	13.1	55	16.5	49	10.7
Missings	0		5		11		9	
**Maternal cotinine level at 3rd trimester**								
< 100 ng/mL	405	73.0	381	84.1	252	80.5	366	87.4
≥ 100 ng/mL	150	27.0	72	15.9	61	19.5	53	12.6
Missings	53		18		32		46	
**Maternal alcohol consumption during pregnancy**								
No	455	74.8	385	79.6	308	89.3	378	81.3
Yes	153	25.2	96	20.4	37	10.7	87	18.7
Missings	0		0		0		0	

Values are percentages for categorical variables and mean (SD) for continuous variables

Supplemental Material, Table 1c. Child and parental characteristics of the study population by region

	Valencia (n=608)		Sabadell (n=471)		Asturias (n=345)		Gizpuzkoa (n=465)	
	n	Distribution	n	Distribution	n	Distribution	n	Distribution
**Maternal fruit & vegetable consumption, 1st trimester**[a]								
≤405 gr/day	241	39.8	155	33.0	105	30.5	126	27.6
>405 gr/day	364	60.2	315	67.0	239	69.5	331	72.4
Missings	3		1		1		8	
**Maternal consumption of large fatty fish at 1st trimester**								
0 servings/week	299	49.4	285	60.6	104	30.2	179	39.2
0-1 servings/week	245	40.5	154	32.8	159	46.2	254	55.6
>1 servings/week	61	10.1	31	6.6	81	23.6	24	5.2
Missings	3		1		1		8	
**Maternal consumption of small fatty fish at 1st trimester**								
0 servings/week	284	49.9	172	36.6	215	62.5	197	43.1
0-1 servings/week	249	41.2	248	52.8	87	25.3	228	49.9
>1 servings/week	72	11.9	50	10.6	42	12.2	32	7.0
Missings	3		1		1		8	
**Maternal consumption of lean fish at 1st trimester**								
0-2 servings/week	253	41.8	137	29.2	75	21.8	99	21.7
2-3 servings/week	95	15.7	63	13.4	63	18.3	75	16.4
>3 servings/week	257	42.5	270	57.4	206	59.9	283	61.9
Missings	3		1		1		8	
**Use of gas stove during pregnancy**								
No	220	36.2	177	38.0	266	79.6	388	85.1
Yes	387	63.8	289	62.0	68	20.4	68	14.9
Missings	1		5		11		9	
**Pre-pregranacy BMI**								
Underweight/Normal weight	435	71.6	343	72.8	240	69.6	380	81.7
Overweight	117	19.2	88	18.7	77	22.3	64	13.8
Obese	56	9.2	40	8.5	28	8.1	21	4.5
Missings	0		0		0		0	
**Maternal working status through child 2nd year of life**								
Working	326	54.1	323	71.2	199	67.2	332	76.0
Not working	277	45.9	131	28.8	97	32.8	105	24.0
Missings	5		17		49		28	
**Maternal TSH level at 1st trimester (mU/l)**	571	1.5 (1.2)	463	1.5 (1.2)	227	1.8 (1.7)	461	1.5 (1.0)
Missings	37		8		118		4	
**Maternal haemoglobin level at 1st trimester (g/dl)**	603	13.1 (0.8)	452	12.7 (0.9)	332	13.0 (0.8)	443	12.9 (0.8)
Missings	5		19		13		22	
**Maternal circulating vitamin D levels at pregnancy**[b]								
Low tertile	130	22.7	185	40.4	131	40.0	161	35.7
Medium tertile	195	34.0	124	27.1	116	35.5	162	35.9
High tertile	248	43.3	149	32.5	80	24.5	128	28.4
Missings	35		13		18		14	

Values are percentages for categorical variables and mean (SD) for continuous variables

[a]Low *vs.* medium/high tertile of maternal fruit & vegetable consumption

[b]Season-specific tertiles of maternal circulating Vitamin D levels (see methods section)

Supplemental Material, Table 2. Details of the imputation modelling

**Software used and key setting:** STATA 10.1 software (Stata Corporation, College Station, Texas) – Ice command (with 10 cycles)
**Number of imputed datasets created:** 20
**Variables included in the imputation procedure:**<br>*Variables used in the main analyses (outcome, exposure, and potential confounders)*<br>Mental development score, sex, gestational age, type of delivery, apgar score at 5th minute, birthweight, birthlength, birth cephalic perimeter, number of siblings at birth, maternal pre-pregnancy weight and height, maternal age, social class, education, and country of birth, paternal age, social class, education, and country of birth, marital status, use of gas stove at home during pregnancy, maternal smoking and alcohol use during pregnancy, maternal tobacco environmental exposure at home during pregnancy, maternal cotinine level at 3rd trimester, maternal diet at 1st trimester of pregnancy (fish consumption, fruits and vegetables, etc.), maternal TSH level at 1st trimester, maternal haemoglobin level at 1st trimester, season-specific maternal circulating vitamin D levels at 1st trimester, noise annoyance at home during pregnancy, maternal hours spend at home at 3rd trimester, cord blood lead concentration, main caregiver through child 2nd year of life, nursery attendance through child 2nd year of life, weeks of any breastfeeding until child 2nd year of life, weeks of exclusive breastfeeding until child 2nd year of life, maternal working status through child 2nd year of life.<br>*Variables only used for imputation models*<br>Maternal working status at 1st and 3rd trimester, age of the house, number of persons living at home, use of extractor fan during pregnancy and through child 2nd year of life, frequency of ventilation of the house during pregnancy and through child 2nd year of life, small for gestational age (weight, length, cephalic perimeter), number at siblings through child 2nd year of life, maternal smoking through child 2nd year of life, child tobacco environmental exposure through child 2nd year of life, use of gas stove at home through child 2nd year of life.
**Treatment of non-normally distributed variables:** log-transformed
**Treatment of binary/categorical variables:** logistic, ordinal, and multinomial models
**Statistical interactions included in imputation models:** none, but we performed multiple imputarion separately by region (Valencia, Sabadell, Asturias, and Gipuzkoa)

Supplemental Material, Figure 1. Bivariate association (β coefficient and 95% of Confidence Interval) between study variables and infant mental development[a].

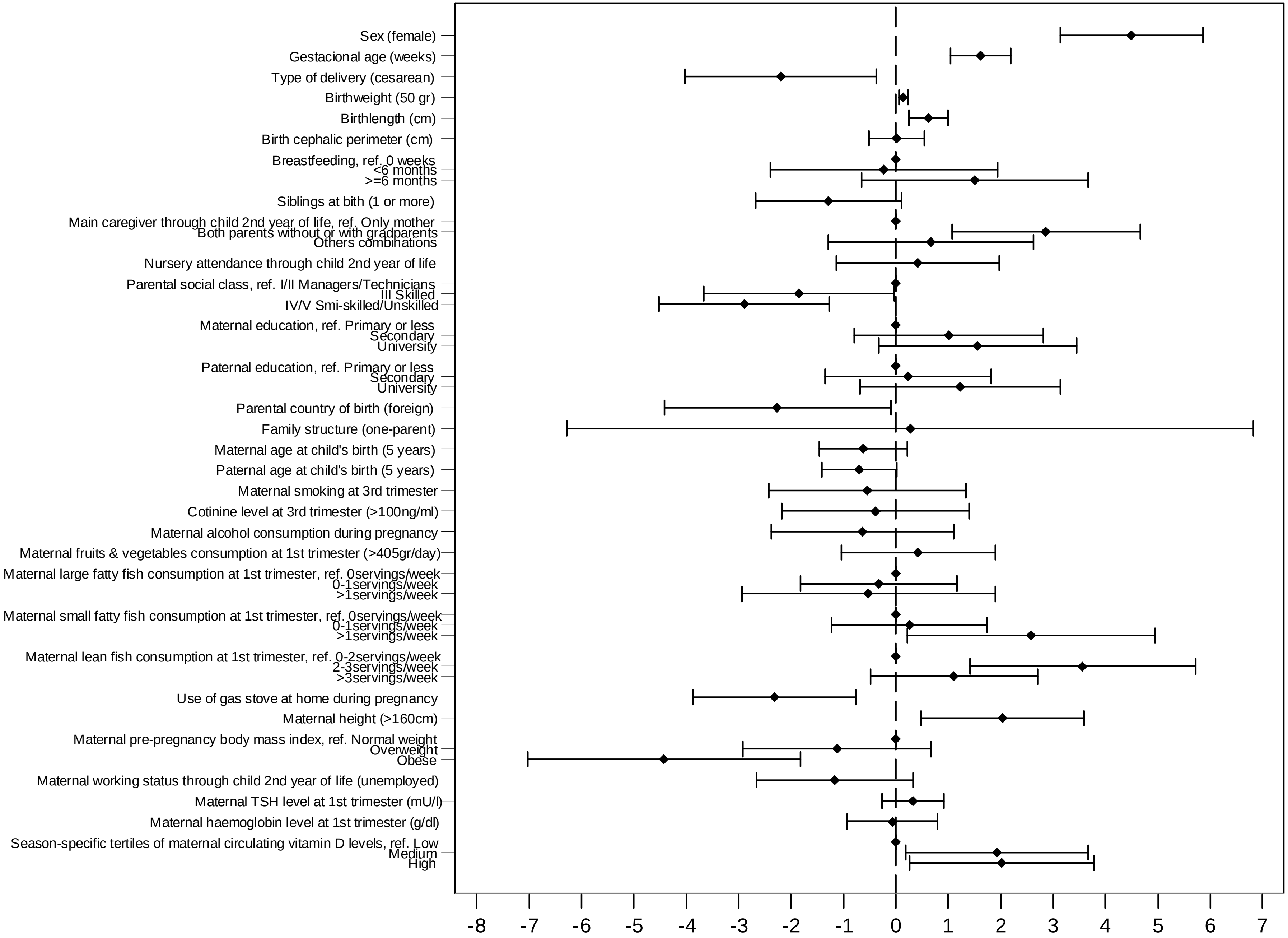

[a]Each models was adjusted for child's age at mental development assessment and region

Supplemental Material, Figure 2. Flowchart illustrating the main phases in the study.

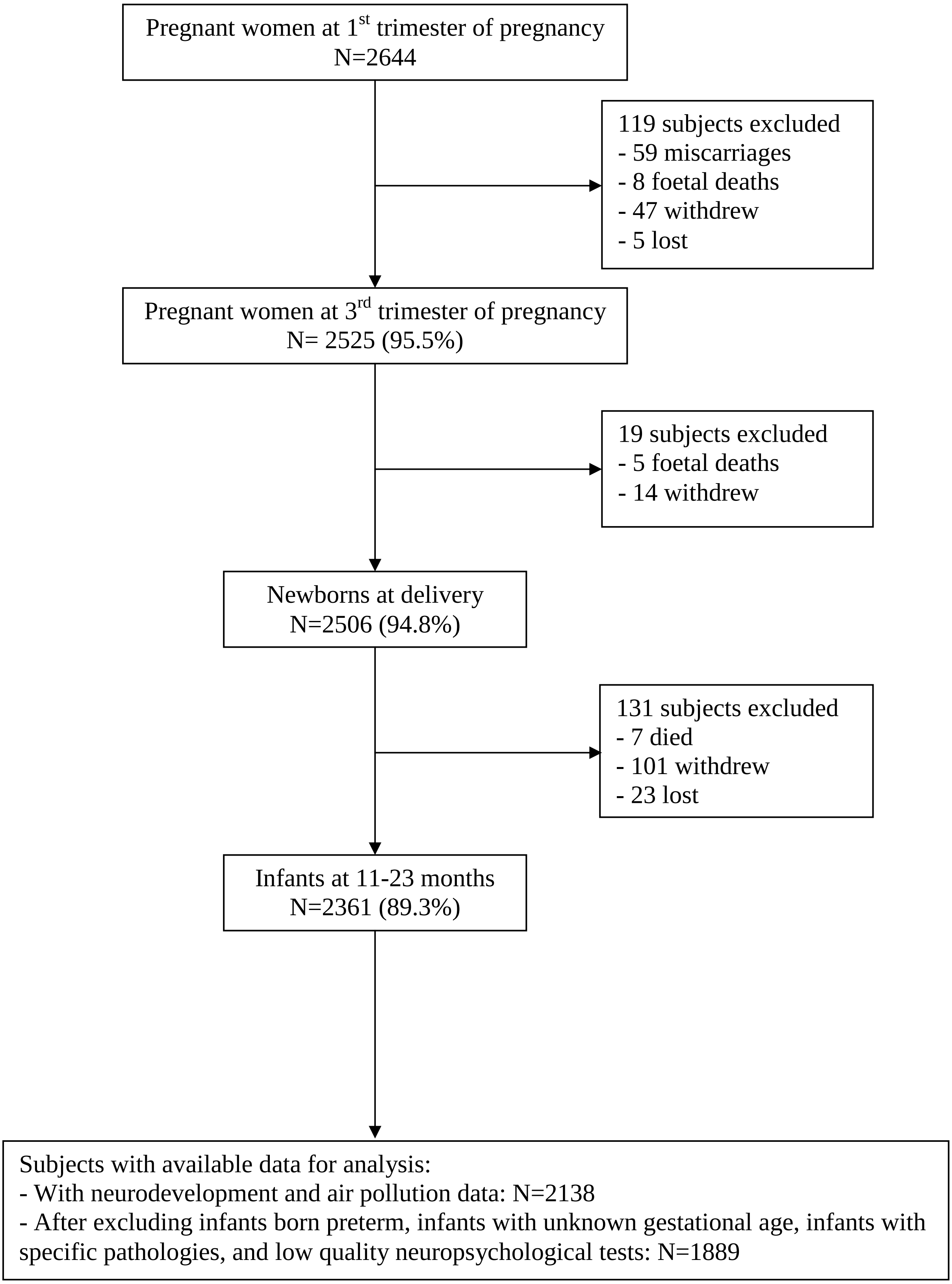

Supplemental Material, Table 3. Differences on child and parental characteristics between participants included and those not included in the present analyses

	Participants included (n=1889)	Participants not included (n=616)	p-value of difference
**Child variables**			
**Sex (female *vs.* male)**	50.0	42.3	0.002
**Gestational age (weeks)**	39.8 ± 1.2	38.8 ± 2.6	<0.001
**Birthweight**	3298 ± 423	3121 ± 602	<0.001
**Breastfeeding duration**			0.011
No	14.7	17.7	
<6months	40.5	45.2	
≥6m	44.9	37.1	
**Number of siblings at child's birth (1 or more *vs.* 0)**	41.9	46.3	0.071
**Parental variables**			
**Parental social class**			0.001
I/II Managers/Technicians	32.8	27.0	
III Skilled	25.7	23.2	
IV/V Semi-skilled/unskilled	41.5	49.8	
**Maternal education level**			<0.001
Primary or less	22.8	29.6	
Secondary	41.2	41.6	
University degree	36.0	28.9	
**Paternal education level**			0.057
Primary or less	34.4	38.6	
Secondary	43.8	43.6	
University degree	21.8	17.8	
**Parental country of birth (foreign *vs.* Spanish)**	7.8	9.3	0.228
**Family status (biparental *vs.* monoparental)**	98.9	96.9	0.001
**Maternal age at child's birth (years)**	31.9 ± 4.2	32.1 ± 4.6	0.006
**Paternal age at child's birth (years)**	34.1 ± 4.9	34.1 ± 5.1	0.311
**Maternal smoking at 3rd trimester (yes *vs.* no)**	16.2	20.0	0.034
**Maternal alcohol use during pregnancy (yes *vs.* no)**	19.8	21.9	0.245
**Use of gas stove during pregnancy (yes *vs.* no)**	43.6	45.4	0.450
**Maternal pre-pregnancy body mass index**			0.850
Normal weight/Underweight	74.0	72.9	
Overweight	18.3	19.2	
Obese	7.7	8.0	

Values are percentages for categorical variables and mean ± standard deviation for continuous variables

Supplemental Material, Figure 3. Region and summary risk estimates (β coefficient and 95% of Confidence Interval) for a doubling in NO_2 and benzene levels during pregnancy and infant mental development by maternal fruits and vegetables consumption levels[a].

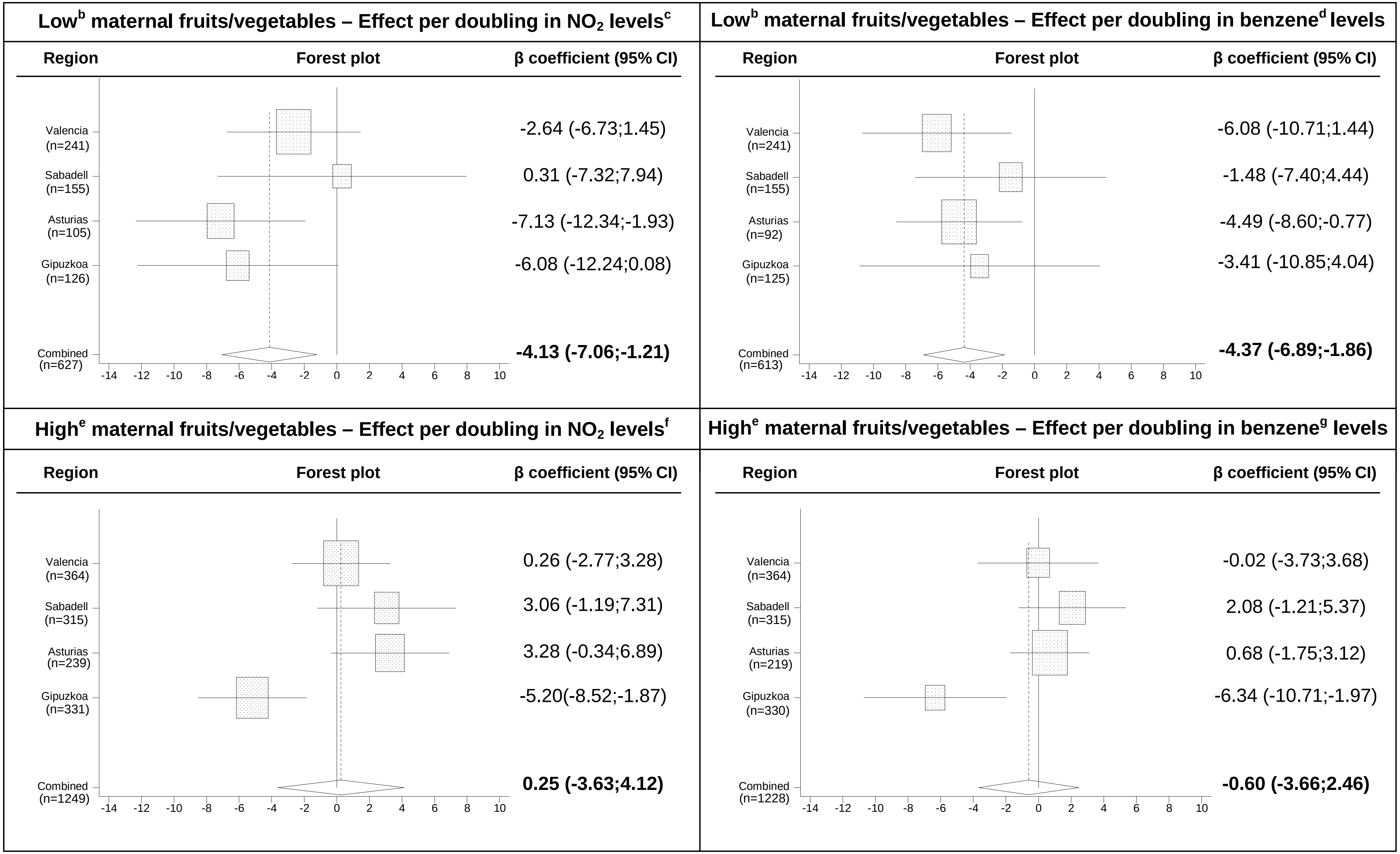

[a]Adjusted for psychologist, child's sex, child's age at mental development assessment, maternal education, maternal age, ,maternal height, and pre-pregnancy body mass index, maternal alcohol use during pregnancy, maternal large fatty and lean fish consumption at 1st trimester, season-specific maternal circulating vitamin D level at pregnancy, use of gas stove at home during pregnancy, and number of siblings at birth
[b]≤405 gr/day
[c]Test for heterogeneity: $Q = 3.472$ on three degrees of freedom ($p = 0.324$)
[d]Test for heterogeneity: $Q = 1.526$ on three degrees of freedom ($p = 0.676$)
[e]>405 gr/day
[f]Test for heterogeneity: $Q = 14.549$ on three degrees of freedom ($p = 0.002$)
[g]Test for heterogeneity: $Q = 9.933$ on three degrees of freedom ($p = 0.019$)
The size of the markers for each β coefficient represents the relative weight each region that contributed to the summary regression slope

Supplemental Material, Table 4. Adjusted association (β coefficient and 95% of Confidence Interval) for a doubling in NO_2 and benzene levels during pregnancy and infant mental development. Pooled analyses excluding Gipuzkoa cohort[a]

	Effect per doubling in NO_2 levels during pregnancy				Effect per doubling in benzene levels during pregnancy			
	**n**	**β**	**(95% CI)**	**p-value interaction**	**n**	**β**	**(95% CI)**	**p-value interaction**
**Maternal fruits/vegetables consumption, 1st trimester[b]**								
≤405 gr/day	501	-2.00	(-4.95;0.96)		488	-3.44	(-6.01;-0.87)	
>405 gr/day	918	2.17	(-0.15;4.18)	0.094	898	1.20	(-0.48;2.88)	0.006
**Breastfeeding duration**								
No	577	-2.54	(-6.70;1.62)		217	-3.78	(-7.25;-0.31)	
<6m	225	1.25	(-1.35;3.85)	0.899	561	0.49	(-1.71;2.68)	0.022
>6m	593	1.75	(-0.91;4.41)	0.733	585	1.05	(-1.38;3.48)	0.040
**Maternal circulating vitamin D levels[c]**								
Low	446	1.01	(-1.78;3.80)		435	-1.13	(-3.45;1.20)	
Medium	435	-0.18	(-3.46;3.09)	0.942	424	0.04	(-2.63;2.72)	0.358
High	477	1.58	(-1.30;4.49)	0.427	469	0.65	(-1.87;3.17)	0.515
**Parental social classs**								
I/II Managers/ Technicians	406	0.97	(-2.08;4.02)		393	-0.45	(-2.87;1.98)	
III Skilled manual/non-manual	389	1.87	(-1.57;5.31)	0.667	380	0.66	(-2.26;3.57)	0.811
IV/V Semi-skilled/ unskilled	628	-0.09	(-2.56;2.39)	0.565	617	-1.32	(-3.50;0.87)	0.422
**Maternal education level**								
Primary or less	370	0.33	(-3.29;3.95)		366	-1.55	(-4.96;1.87)	
Secondary	611	-0.49	(-2.93;1.95)	0.193	600	-0.68	(-2.69;1.33)	0.287
University degree	440	1.65	(-1.39;4.68)	0.608	422	-0.28	(-2.77;2.21)	0.189

[a]Adjusted for region, child's sex, child's age at mental development assessment, maternal education, maternal age, ,maternal height, and pre-pregnancy body mass index, maternal alcohol use during pregnancy, maternal large fatty and lean fish consumption at 1st trimester, season-specific maternal circulating vitamin D level at pregnancy, use of gas stove at home during pregnancy, and number of siblings at birth.
[b]Low *vs.* medium/high tertile of maternal fruit & vegetable consumption.
[c]Season-specific tertiles of maternal circulating vitamin D levels (see methods section). These models were not adjusted for maternal vitamin D levels at pregnancy.

Supplemental Material, Table 5. Characteristics of each region

Regions	Area (km^2)	Number munici-palities	Description	Type of industry	NO_2 levels ($\mu g/m^3$) (mean±SD)	Benzene levels ($\mu g/m^3$) (mean±SD)	Correlation coefficient NO_2 *vs.* benzene	$PM_{2.5}$[a-c] (range in $\mu g/m^3$)	Trace elements metals[a-c] (range in ng/m^3)	% > LOQ lead levels in cord blood[d]
Valencia	1372	32	Urban + metropolitan + semi-urban + rural	None	36.8 ± 11.0	2.2 ± 0.6	0.69	18.0-24.0	Pb: 5.9-18.9<br>Mn: 2.4-3.9	6.6
Sabadell	38	1	Urban	None	32.1 ± 8.8	0.8 ± 0.3	0.75	10.4-32.4	Pb:13.1-22.4<br>Mn:9.0-13.1	2.3
Asturias	483	9	Urban-industrial + semi-urban + rural	Steelworks, glassworks, chemical	23.2 ± 7.1	2.3 ± 1.3	0.61	10.4-24.1	Not measured	4.1
Gipuzkoa	519	26	3 narrow and uneven valleys - Industrial zone	Iron and steel industry sector	20.1 ± 6.5	1.0 ± 0.3	0.71	12.5-28.9	Pb:20.3-224.0<br>Mn:10.2-124.0	8.7

GM=Geometric mean; LOQ=Limit of quantification (2 µg/dl); Mn=Manganese; Pb=Lead; SD=Standard deviation

[a]Rivas-Lara I. 2008. Variabilitat temporal i geogràfica i caracterització química de la contaminació atmosfèrica particulada a Sabadell [in Catalan] [MSc thesis] Barcelona, Spain: Autonomous University of Barcelona. Available: http://www.recercat.net/bitstream/2072/12571/1/PFC+Ioar+Rivas.pdf [accessed 20 January 2011]

[b]Viana M, Querol X, Alastuey A, Ballester F, Llop S, Esplugues A, et al. 2008. Characterising exposure to PM aerosols for an epidemiological study. Atm Env. 42:1552-1568.

[c]Lertxundi A, Martinez MD, Ayerdi M, Alvarez J, Ibarluzea JM. 2010. Air quality assessment in urban areas of Gipuzkoa (Spain). Gac Sanit. 24:187-192.

[d]Llop S, Aguinagalde X, Vioque J, Ibarluzea J, Guxens M, Casas M, et al. 2011. Prenatal exposure to lead in Spain: Cord blood levels and associated factors. Sci Total Environ. 409(11):2298-305
